# Integrative analysis of disulfidptosis and immune microenvironment in hepatocellular carcinoma: a putative model and immunotherapeutic strategies

**DOI:** 10.3389/fimmu.2023.1294677

**Published:** 2024-01-03

**Authors:** Ti Yang, Junhao Liu, Fang Liu, Jiashun Lei, Siliang Chen, Zengxin Ma, Peifeng Ke, Qiaolan Yang, Jianfan Wen, Yifeng He, Juan Duan, Xiancheng Zeng

**Affiliations:** ^1^ Department of Hepatobiliary-pancreatic&hernia Surgery, Guangdong Second Provincial General Hospital, Guangzhou, Guangdong, China; ^2^ The Second School of Clinical Medicine, Southern Medical University, Guangzhou, Guangdong, China; ^3^ The First School of Clinical Medicine, Southern Medical University, Guangzhou, Guangdong, China; ^4^ Department of Interventional Radiology, Guangdong Second Provincial General Hospital, Guangzhou, Guangdong, China; ^5^ Guangdong Provincial Hospital of Chinese Medicine, The Second Affiliated Hospital of Guangzhou University of Chinese Medicine, Guangzhou, Guangdong, China; ^6^ Department of General Management, Guangdong Second Provincial General Hospital, Guangzhou, Guangdong, China

**Keywords:** HCC, disulfidptosis, putative model, immune microenvironment, NDRG1

## Abstract

**Background:**

Hepatocellular carcinoma (HCC) is a malignant tumor with a high rate of recurrence and m metastasis that does not respond well to current therapies and has a very poor prognosis. Disulfidptosis is a novel mode of cell death that has been analyzed as a novel therapeutic target for HCC cells.

**Methods:**

This study integrated bulk ribonucleic acid (RNA) sequencing datasets, spatial transcriptomics (ST), and single-cell RNA sequencing to explore the landscape of disulfidptosis and the immune microenvironment of HCC cells.

**Results:**

We developed a novel model to predict the prognosis of patients with HCC based on disulfidptosis. The model has good stability, applicability, and prognostic and immune response prediction abilities. N-myc downregulated gene1 (NDRG1) may contribute to poor prognosis by affecting macrophage differentiation, thus allowing HCC cells to evade the immune system.

**Conclusion:**

Our study explores the disulfidptosis of HCC cells through multi-omics and establishes a new putative model that explores possible targets for HCC treatment.

## 1 Introduction

Liver cancer is the third most common cause of cancer deaths worldwide, accounting for 8.3% of the overall cancer mortality (1). Hepatocellular carcinoma (HCC) accounts for about 85%-90% of all primary liver malignancies (2). Although new therapies have emerged, like immunotherapy, targeted therapy, and radiation therapy, the five-year survival of advanced HCC is less than 15% (3). New immune checkpoint inhibitors, such as Nivolumab, Atezolizumab, and Ipilimumab, are currently on the market, but their therapeutic efficacy is not promising, possibly due to immune escape (4). A lack of effective treatment has prompted a search for new biomarkers.

Programmed cell death is associated with numerous pathophysiological processes, including tumor progression and alterations in the surrounding immune microenvironment (5). Several new cell death models have recently arisen: apoptosis, cuproptosis, ferroptosis, necroptosis, lysosome-dependent cell death, immunogenic cell death, and autophagy-dependent cell death (6, 7). Liu et al. recently discovered a new mode of cell death: disulfidptosis. In glucose-starved cells overexpressing solute carrier family 7 member 11 (SLC7A11), disulfidestress caused by excessive intracellular cystine accumulation can cause rapid cell death (8). Normal disulfide bonds between cytoskeletal proteins are disrupted by accumulation of disulfide material, leading to collapse of the histone skeleton and cell death. Glucose transporter inhibitors trigger disulfidptosis and suppress tumor proliferation.

The bulk ribonucleic acid (RNA) sequencing is the average messenger RNA (mRNA) expression in all cells, which does not reflect the state of single cells in the tissue. Single-cell RNA sequencing (scRNA-seq) enables a detailed analysis of the tumor microenvironment heterogeneity at the single-cell resolution level (9, 10). However, scRNA-seq fails to preserve the tissues’ spatial structures. The complicated cellular interactions that transpire across the entire tissue space cannot be accurately deciphered. The advent of spatial transcriptomics (ST) technology facilitates the spatial exploration of gene expression and preserves cell arrangements during multicellular tissue analysis. Thus, combining single-cell technology with ST may detect details regarding heterogeneous cell populations and provide insight into spatial tissue organization (11, 12).

**Figure 1:**
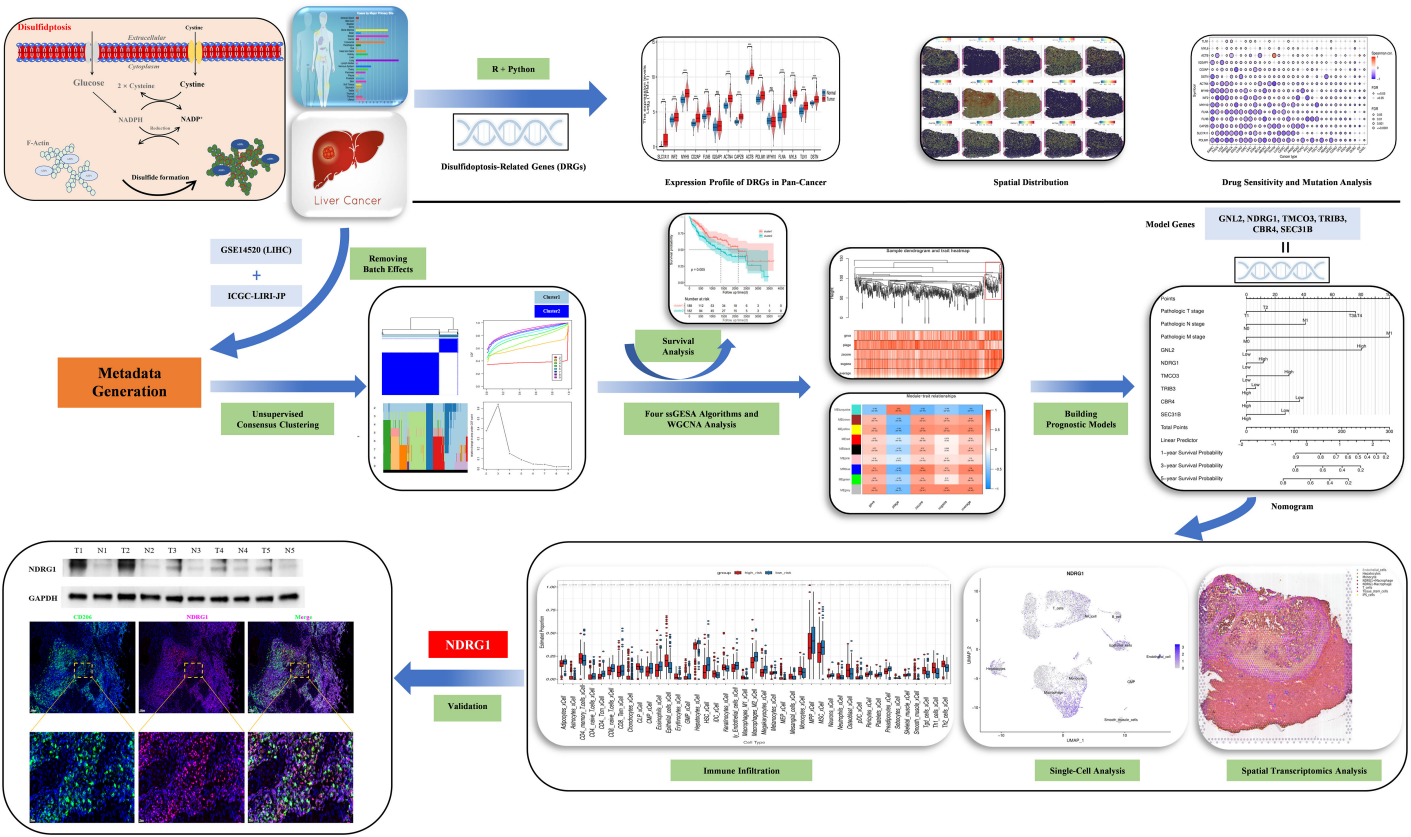
Study flow chart.

In our study, we employed a multi-omics strategy to investigate the landscape ofdisulfidptosis in HCC. We constructed a survival prognostic model using bulk RNA sequencing and confirmed the model has good prognostic and immune response prediction abilities. Importantly, our findings revealed elevated expression levels of N-myc downregulated gene1 (NDRG1) was expressed more in tumor macrophages and promoted Polarization of M2-type macrophages. These findings provide a theoretical basis for exploring effective biomarkers in HCC and improving the efficacy of anti-tumor immune therapy. Outline of the study design is shown in **Figure 1**.

## 2 Results

### 2.1 The expression of disulfidptosis related genes in pan-cancer

**Figure 2:**
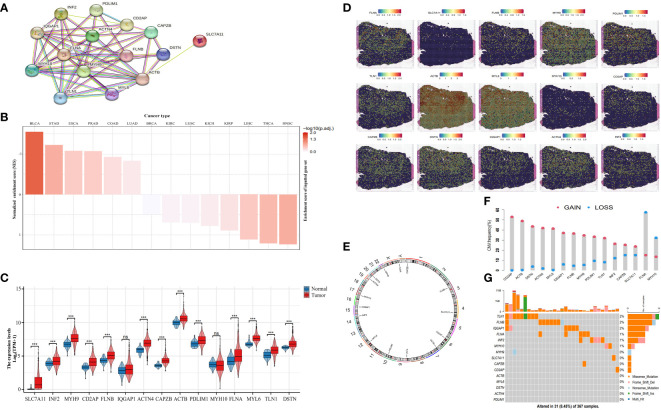
Landscape of DRGs in pan-cancer. **(A)** The correlation network of the 15 DRGs. **(B)** The enrichment score of DRGs in pan-cancer. **(C)** The different expression of DRGs between HCC and normal tissue. **(D)** Spatial expression levels of DRGs in HCC tissue sections. **(E)** The location of the CNV alteration of the changes in DRGs in 23 chromosomes. **(F)** The frequency of CNV variation in DRGs (blue: CNV deletion; red: CNV amplification). **(G)** Analysis of SNV in DRGs. ns, not statistically significant; *P< 0.05; **P< 0.01; ***P< 0.001; ****P< 0.0001.

We established a protein-protein correlation network with 15 DRGs (**Figure 2A**), which was derived from previous research (8) and identified SLC7A11 as a key player within this network. Furthermore, we employed the Single-sample Gene Set Enrichment Analysis (ssGSEA) algorithm to assess the disulfidptosis score in pan-cancer. The results revealed a positive normalized enrichment score, indicating upregulation of disulfidptosis. In bladder urothelial carcinoma (BLCA), the normalized enrichment score was negative and disulfidptosis was downregulated (**Figure 2B**). In HCC, the expression levels of SLC7A11, inverted formin 2 (INF2), myosin heavy chain 9 (MYH9), CD2 associated protein (CD2AP), filamon B (FLNB), actinin alpha 4 (ACTN4), capping actin protein of muscle Z-line subunit beta (CAPZB), actin B (ACTB), PDZ and LIM domain 1 (PDLIM1), filamin A (FLNA), myosin light polypeptide 6 (MYL6), talin 1 (TLN1), and destrin (DSTN) were remarkably higher than the normal tissues. MYH10 and Ras GTPase-activating-like protein (IQGAP1) (**Figure 2C**) were not higher, although this difference was not statistically significant. We then evaluated the expression of DRGs in tissue sections using ST analysis. Generally, DRGs were highly expressed around and in tumors, except for MYH9 and MYH10 (**Figure 2D**). To investigate their mutations, we downloaded copy number variation (CNV) and single nucleotide variants (SNV) data from the The Cancer Genome Atlas (TCGA) database. **Figure 2E** displays the positions of CNV changes in DRGs on their corresponding chromosomes. Despite the high frequency of deletions in MYH10 and FLNA, CNVs were still common and mostly involved in amplification (**Figure 2F**). We then analyzed the prevalence of SNV in 15 DRGs and found that 31 (8.54%) of 367 liver hepatocellular carcinoma (LIHC) samples showed mutations in the DRGs. Among them, IQGAP1, FLNB, and TLN1 had the maximum mutation frequency (2%), followed by MYH10, INF2, and FLN1, while others displayed no obvious mutations (**Figure 2G**). Thus, our results suggest that DRGs may act in pan-cancer onset and progression.

### 2.2 Methylation levels and drug sensitivity of DRGs

**Figure 3:**
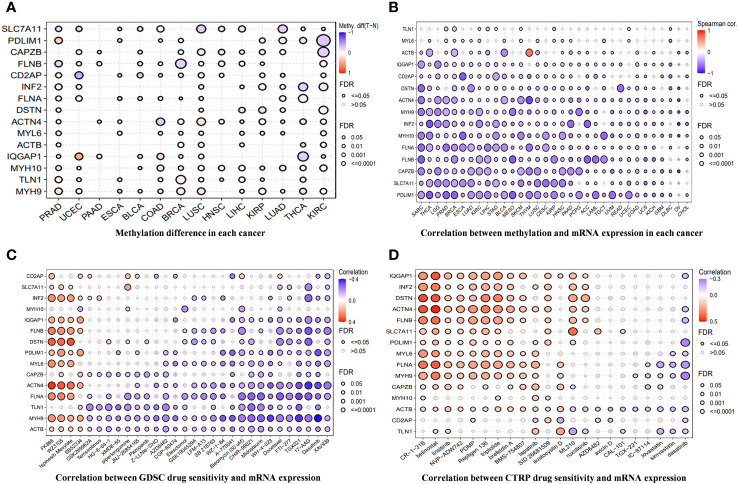
**(A)** The differences in methylation of DRGs in each cancer. **(B)** The correlation between methylation across different cancer types and the mRNA expression of DRGs. **(C, D)** A drug-sensitive analysis aimed at DRGs based on GDSC and CTRP.

**Figure 3A** shows the methylation levels of DRGs in pan-cancer. CD2AP had the lowest methylation level in uterine corpus endometrial carcinoma (UCEC), and IQGAP1 had the highest methylation level in UCEC. Except for ACTB in Thymoma and Ovarian Cancer, the methylation levels of DRGs in pan-cancer had different degrees of negative correlation with mRNA expression (**Figure 3B**). Drug sensitivity prediction against DRGs using two drug sensitivity databases revealed that the drugs with the strongest predictive sensitivity in the GDSC were FK866, WZ3105, Ispinesib Mesylate, and SB52334. In the Cancer Therapeutics Response Portal (CTRP) database, the drugs with strong predictive sensitivity were CR-1-31B, belinostat, Palmitoyl-DL-carnitine hydrochloride (PDMP), Repligen 136, and triptolide (**Figures 3C, D**).

### 2.3 Identification and exploration of DRGs in HCC

**Figure 4:**
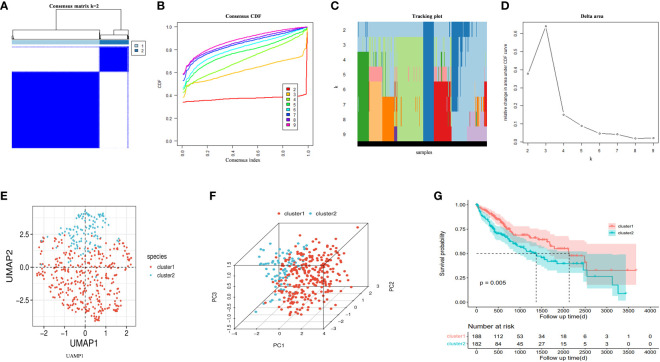
**(A)** An unsupervised consensus clustering heatmap. **(B)** The plot of the relative area changes from k = 2 to 9 under the cumulative distribution function (CDF) curve. **(C)** Consistent CDF plot. **(D)** Tracing plot of the clustered samples. **(E)** Principal Component Analysis. **(F)** Uniform Manifold Approximation and Projection Analysis. **(G)** The OS curves between clusters.

An unsupervised consistent cluster analysis of patients with HCC based on the expression of DRGs yielded two disulfidptosis subgroups (**Figures 4A–D**). We performed principal component analysis (PCA) and uniform manifold approximation and projection (UMAP) analyses and observed that the two clusters were separated in space (**Figures 4E, F**). Survival analyses for both groups of patients indicated a significant difference in their survival time (**Figure 4G**), with cluster1 showing a better prognosis. Similar clustering modes were noted in the TCGA dataset (**Supplementary Figure 1A–D**). The results of different datasets were highly consistent, further demonstrating the reliability and stability of our typing.

### 2.4 Disulfidptosis score and weighted gene co-expression network analysis

**Figure 5:**
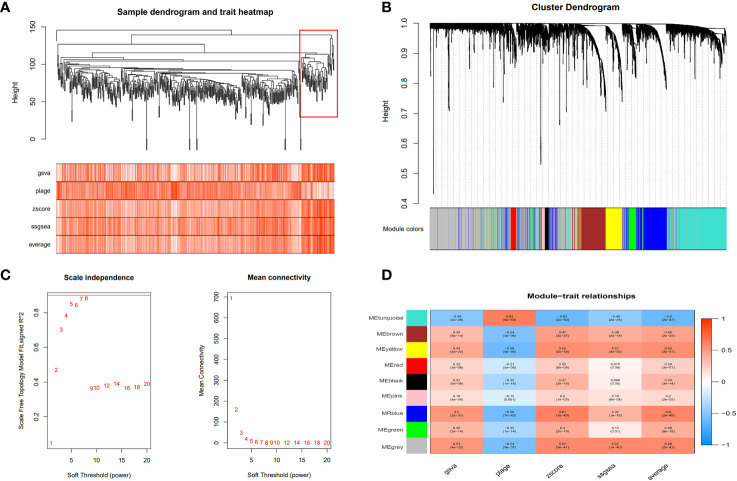
Weighted gene co-expression network analysis (WGCNA). **(A)** The cluster dendrogram of co-expression genes in HCC. The red boxes are dendrogram regions corresponding to disulfidptosis. **(B)** Cluster analysis of HCC samples to detect outliers (the white-to-red linear gradient color is associated with the disulfidptosis score, and the grey color indicates missing data). **(C)** Determination of soft-thresholding power in the WGCNA. **(D)** Module–trait relationships in HCC. Each cell contains the corresponding correlation and P-value.

We utilized the “gene set variation analysis (GSVA)” R package to apply the “gsva,” “plage,” “zscore,” and “ssgsea” algorithms to score gene expression in the metadata associated with disulfidptosis. The average value of these scores was calculated. Pearson’s correlation method and the mean linkage method were employed to correlate the dendrograms of the samples with disulfidptosis score traits (**Figure 5A**). To construct co-expression networks, we performed co-expression analysis with a soft threshold of 18 (scale-free R^2 =^ 0.9) to ensure a scale-free network. The dendrograms of all differentially expressed genes were clustered based on the differential measure (1-TOM) (**Figures 5B, C**). Through hierarchical clustering, a total of nine units were identified. Among these units, we selected the blue module, which exhibited the highest correlation with the disulfidptosis score, as the clinically significant module for further analysis. Within the blue module, we identified 753 phenotypic genes (**Figure 5D**).

### 2.5 Construction and verification of the blue module-based prognostic signature

**Figure 6:**
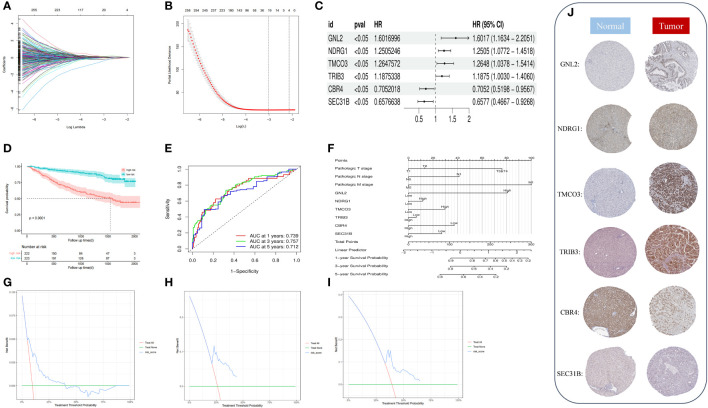
**(A)** Identification of optimal variables through LASSO regression with 10-fold cross-validatio. **(B)** The solution path was plotted according to coefficients against the L1 norm. **(C)** The forest plot shows the hazard ratios and 95% confidence intervals of signature genes from the multivariate Cox regression analysis. **(D)** We plotted the OS curves between high-risk and low-risk groups based on the prognostic signature. **(E)** The time-dependent ROC curves. **(F)** We constructed a nomogram model to predict the one-year, three-year, and five-year OS of HCC patients. **(G–I)** DCA for the one-year **(G)**, three-year **(H)**, and five-year **(I)** OS of the risk model. **(J)** Representative immunohistochemical staining images of GNL, NDRG1, TMCO3, TRIB3, CBR4, and SEC31B in normal and tumor tissues were retrieved from the HPA (https://www.proteinatlas.org/, accessed: January 2023).

We performed a univariate Cox regression analysis on 753 phenotypic genes and screened 507 candidate genes with prognostic values (**Supplementary Figure·2**). After performing a LASSO regression analysis (**Figures 6A–C**) and multivariate Cox regression analysis, we obtained a six-gene model. G protein nucleolar 2 (GNL2), NDRG1, transmembrane and coiled-coil domains 3 (TMCO3), tribbles pseudokinase 3 (TRIB3), carbonyl reductase 4 (CBR4), and SEC31 homolog B, COPII coat complex component (SEC31B) were the prognostic indicators for establishing a risk model with a C-index of 0.717. Based on the median risk score, we classified patients into low- (n = 223) and high-risk groups (n = 222).

We plotted and compared survival analysis and recipient work characteristic curves to determine the accuracy of the prognostic characteristic predictions. According to the Kaplan-Meier analysis, overall survival (OS) was considerably longer in the low-risk group versus the high-risk group (**Figure 6D**). The results of the study indicated that in the metadata cohort, the area under the receiver operating characteristic (ROC) curve of the risk model for one-, three-, and five-year OS was 0.739, 0.757, and 0.712, respectively. (**Figure 6E**). According to the decision curve analysis (DCA), the risk model predicted favorable net clinical benefits for OS at one, three, and five years in patients with HCC (**Figures 6G–I**). To further verify the model’s general applicability, we conducted DCA analysis, ROC analysis, and Kaplan-Meier analysis on the model with the validation set TCGA-LIHC and GSE144269. The validation TCGA-LIHC process demonstrated the model’s robustness and applicability. Notably, the survival analysis conducted using the model revealed a significant difference in survival between the high and low-risk groups (P = 0.00091) (**Supplementary Figure 1E**). Furthermore, the model’s performance was evaluated using ROC curves in the validation set. The area under the curve (AUC) values for 1, 3, and 5 years were determined to be 0.721, 0.650, and 0.656 (**Supplementary Figure 1F**), respectively. These results indicate the model’s ability to accurately predict patient outcomes. Additionally, the decision curve analysis demonstrated that the model can provide substantial net clinical benefits in the validation set (**Supplementary Figure 1G**). To further validate the model’s effectiveness, we obtained a new dataset, GSE144269, from the Gene Expression Omnibus (GEO) and conducted another round of validation. Remarkably, the results from this validation set confirmed the previous findings, showing a significant difference in survival between the high and low-risk groups (P = 0.029) (**Supplementary Figure 1H**). The ROC curves for the model in this validation set yielded AUC values of 0.681, 0.644, and 0.586 for predicting patient survival at 1, 3, and 5 years (**Supplementary Figure 1I**), respectively. Moreover, the DCA decision curve analysis indicated that the model can provide substantial net clinical benefits to patients (**Supplementary Figure 1J**). Overall, these validation efforts reinforce the reliability and clinical utility of the prognostic model.

By drawing forest plots of the multifactorial Cox regression analysis (**Figure 6C**), we identified SEC31B and CBR4 as the prognostic protective factors for HCC, whereas the other prognostic markers were risk factors. To characterize the protein expression levels of the signature gene in patients with HCC, we compared the protein expression profiles identified via immunohistochemical staining in the HPA database. These findings suggest that four of the factors in the prognostic profile (NDRG1, GNL2, TRIB3, and TMCO3) were overexpressed in HCC tissues (**Figure 6J**). High expression of SEC31B and CBR4 indicates a positive prognosis for HCC patients. We included pathologic staging in the risk score model and developed a nomogram model to predict one-, three-, and five-year OS (**Figure 6F**). These findings indicate that the model has favorable discriminatory power.

### 2.6 Tumor immune infiltration and GSVA analyses

**Figure 7:**
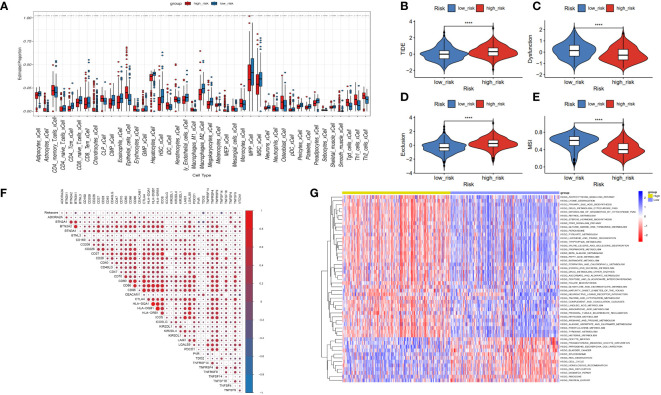
Correlation between risk model and immune function. **(A)** The box diagram displays the difference in immune infiltration between the high-risk and low-risk groups via the Cibersort Algorithm. **(B–E)** Response to immunotherapy in high- and low-risk patients. TIDE score, Dysfunction score, Exclusion score, MSI score. **(F)** Comparison of the expression relevance between risk score and immune checkpoint genes. **(G)** Comparison of the GSVA between the high- and low-risk groups.

To investigate the immune status of various risk groups and their immunotherapy response, we examined the association between risk models and infiltrating immune cells. We assessed differences in the immune status between risk groups by applying the “xCell” and inverse convolution algorithms. The high-risk group had relatively higher levels of Th2 cells, Th1 cells, iDC, neutrophils, Macrophages_M1 cells, and CD4 memory T cells. Levels of the CD8 naive T cells, CD4 Tcm, CD4 naive T cells, Macrophages M2, and CD8 Tem cells were lower (**Figure 7A**).

The tumor immune dysfunction and exclusion (TIDE) scores (**Figure 7B**, P< 0.0001) and exclusion scores (**Figure 7D**, P< 0.0001) were notably higher, and the dysfunction scores (**Figure 7C**, P< 0.0001) and microsatellite instability (MSI) scores (**Figure 7E**, P< 0.0001) were lower in the high-risk group versus the low-risk group. These outcomes suggest that risk scores in patients with HCC may indicate lower immune checkpoint blockade therapy efficacy. High-risk patients may become resistant to immunotherapy. Risk score may potentially be associated with overexpression of other immune checkpoint genes (ICGs), rather than the well-known PD-1 or CTLA4. **Figure 7F** shows the positive association between risk scores and CD209, CD47, CD86, LGALS9, TNFSF4, and TNFSF9. There was a negative association between risk and TDO2, and TNFSF14. Moreover, GSVA analysis indicated that the high-risk group had increased “HOMOLOGOUS_RECOMBINATION,” “MISMATCH_REPAIR,” “RNA_DEGRADATION,” and “RNA_DEGRADATION” pathways (**Figure 7G**).

### 2.7 ScRNA and pseudotime analyses

**Figure 8:**
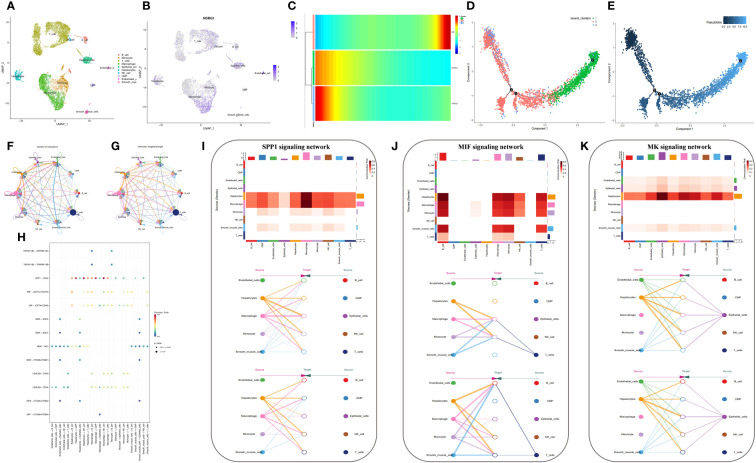
Analysis based on the GSE166635 dataset. **(A)** A UMAP of GSE166635. **(B)** The expression of NDRG1 in different cell clusters. **(C)** The trajectory inference was obtained by a monocle using the RNA velocities method. **(D, E)** A pseudotime analysis of macrophages. These graphs contain cells displayed in a trajectory dimensionality reduction algorithm, colored by group. **(D)** The heatmap showed gene expression (evaluated by the Z-value) as the transition during pseudotime dynamics. **(E)** The circle plots show the overview of cell-cell interaction numbers among cells. **(F)** The circle plot also shows the interaction strength among cells. **(G)** In the tumor tissues, broader arrows indicate stronger interactions. **(H)** An overview of cell-cell communication at the legend-receptor level. **(I-K)** A heatmap shows communication between different cell types in SPP1 **(I)**, MIF **(J)**, and MK **(K)** tumor signals. The hierarchy plots of the SPP1 **(I)**, MIF **(J)**, and MK **(K)** signaling pathway network show the sources and targets.

We generated 21 subgroups through UMAP-based hierarchical clustering of GSE166635 and performed cell annotation using the “singleR” R package, resulting in the identification of 10 distinct cell subgroups (**Figure 8A**). By examining the expression of six genes across various cell types, we observed a significant differential expression of NDRG1 specifically in macrophages (**Figure 8B**). Furthermore, employing RNA rate-based trajectory analysis, we discovered that macrophages in GSE166635 differentiated into two distinct types. Notably, cluster 1 exhibited an initial high expression of NDRG1, as evident from the gradient heatmap (**Figure 8C**). The trajectory diagram (**Figures 8D, E**) revealed that cluster 1 differentiated into a subtype of macrophages. These findings suggest that the elevated expression of NDRG1 contributes to the polarization of macrophages.

**Figure 9:**
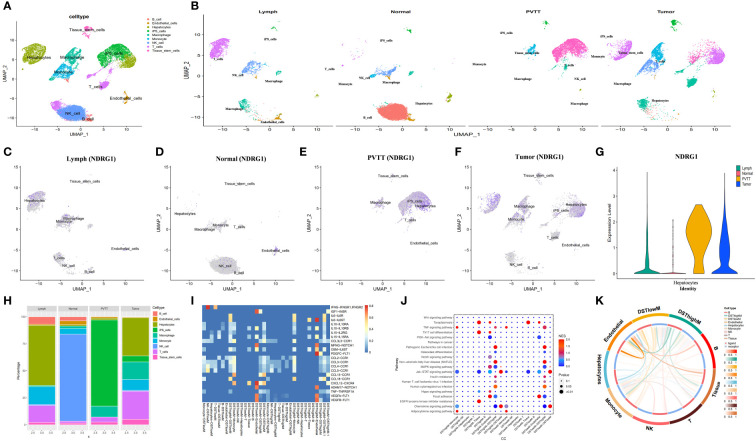
Analysis based on the GSE149614 dataset. **(A)** The UMAP of GSE149614. **(B)** The UMAP of GSE149614 is split by tissues. **(C–G)** The expression of NDRG1 in different tissues. **(H)** Cell infiltration level in various tissues inferred by scRNA-seq. **(I)** Heatmap of ligand-receptor pairs of immune pathways among different cell types in tumor tissues. **(J)** Dot plot of signaling pathways among different cell types in tumor tissues. **(K)** Circle plots showing the overview of cell-cell interaction numbers in tumor tissues.

To explore the immune landscape of disulfidptosis in different tissues, we selected the GSE149614 dataset. Following strict quality control, we analyzed samples from advanced patients and performed UMAP-based hierarchical clustering, resulting in the identification of 15 cell subgroups. Using the “singleR” R package and CellMarker, we annotated these subgroups as “NK cells, B cells, Endothelial cells, T cells, Tissue stem cells, Monocytes, Macrophages, Hepatocytes, and induced pluripotent stem (iPS)” (**Figures 9A, B**). Analysis of cell ratios in different tissue sources from patients with advanced HCC revealed that natural killer (NK) cells were predominant in normal tissues, while hepatocytes, monocytes, T cells, and iPS cells were predominantly present in tumor tissues. These results reflect the malignant, highly differentiated, and immune infiltrative characteristics of tumors (**Figure 9H**). Notably, NDRG1 exhibited differential expression across different tissues, with minimal expression in any cell subtypes of normal tissues and higher expression in macrophages of tumor tissues, lymphoid tissues, and portal carcinoma plugs (**Figures 9C–G**). These findings further validate our observations in GSE166635.

### 2.8 Cell-to-cell communication

Conventional bulk RNA sequencing data is limited in its ability to analyze cellular communication between different cell types. To overcome this limitation, we employed the “cellchat” R package to investigate the pathways involved. Our analysis revealed replicative crosstalk between cells in the GSE166635 dataset (**Figures 8F–H**). Notably, hepatocytes and macrophages exhibited close intercellular connections and sent secreted phosphoprotein 1 (SPP1) signals to nearly all other cell types (**Figure 8I**). Additionally, hepatocytes, macrophages, monocytes, smooth muscle cells, and T-cells transmitted macrophage migration inhibitory factor (MIF) signals to monocytes, macrophages, and B cells (**Figure 8J**). Furthermore, endothelial cells, epithelial cells, and hepatocytes conveyed Midkine (MK) signals to all other cells (**Figure 8K**).

Next, we aimed to investigate the differential expression of NDRG1 in macrophages and its role in macrophage activation during cellular communication. To achieve this, we utilized the “AUCell,” “UCell,” “singscore,” “ssgsea,” and “AddModuleScore” algorithms (implemented through the “AUCell,” “UCell,” “irGSEA,” and “GSVA” R packages) to compute disulfidptosis scores for advanced tumor tissues in the GSE149614 dataset. Subsequently, we classified the macrophages within the tumor tissue based on the median disulfidptosis score, resulting in two groups: Disulfidptosis score high macrophages (DSThighM) and Disulfidptosis score low macrophages (DSTlowM). DSThighM macrophages exhibited a close association with endothelial cells in terms of IL6-IL6ST, MFNG-NOTCH1, OSM-IL6ST, ADAM17-NOTCH1, VEGF1-FLT1, VEGF2-FLT1, and PDGFC-FLT1 ligand-receptor linkages. Furthermore, DSThighM macrophages and endothelial cells displayed a tight relationship in IGF1-INSR, CXCL12-CXCR4, and MFNG-NOTCH1 ligand-receptor pairs (**Figure 9I**). In terms of signaling pathways, DSThighM macrophages and hepatocytes exhibited high activity in Toxoplasmosis, Th17 cell differentiation, and EGFR tyrosine kinase inhibitor resistance. Conversely, the MAPK signaling pathway and focal adhesion were highly active in DSThighM macrophages and endothelial cells. Pathogenic Escherichia coli infection, non-alcoholic fatty liver disease, and human cytomegalovirus infection were closely associated with the autocrine level of DSThighM macrophages. Moreover, the Janus kinase (JAK)-signal transducer and activator of transcription (STAT) signaling pathway and chemokine signaling pathway were highly active in both DSThighM and DSTlowM macrophages (**Figure 9J**). **Figure 9K** illustrates the strength of cellular connections among different cell types.

### 2.9 Disulfidptosis landscape at the spatial transcriptome level

**Figure 10:**
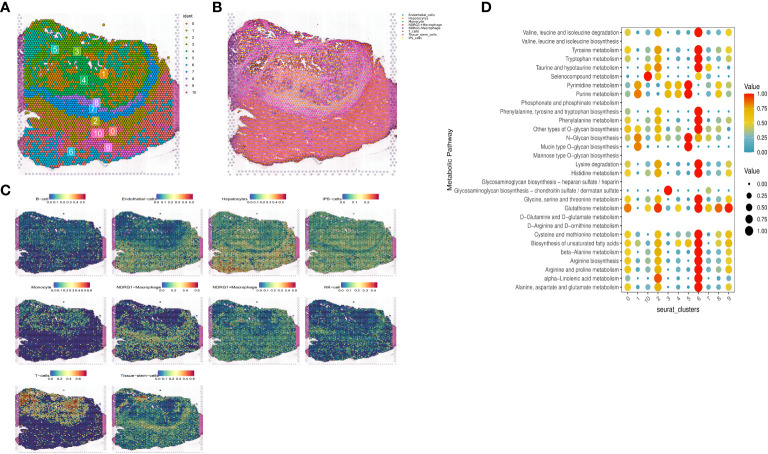
Expression of selected genes in the tissue sections. HE-stained images of HCC tissue sections labeled with eight cell clusters. **(A)** Dimensionality reduction clustering of spots on tissue slices. **(B)** Spatial distribution and expression levels of different cell types on tissue sections. **(C)** Spatial expression levels of different cell types in HCC tissue sections. **(D)** The metabolic status of different cell clusters.

Based on the expression or non-expression of NDRG1, we classified macrophages into two groups: NDRG1+Macrophages and NDRG1-Macrophages. To annotate the spatial patches, we examined the DRGs of each cluster and HE-stained sections, resulting in the identification of eight major clusters: tissue stem cells, endothelial cells, monocytes, T-cells, iPS cells, hepatocytes, NDRG1-Macrophage cells, and NDRG1+Macrophage cells (**Figures 10A, B**). Notably, T-cells, monocytes, and macrophages exhibited increased accumulation within tumors. Comparatively, NDRG1+Macrophage cells were predominantly located in the tumor center, indicating that this subpopulation has a tendency to target the tumor center through chemotaxis. This observation, combined with previous single-cell typing, supports the notion that NDRG1+Macrophage subpopulations specifically migrate towards the tumor center (**Figure 10C**). To assess active metabolism within the tumor region at the ST level, we employed the “scMetabolism” R package, revealing metabolic activity patterns (**Figure 10D**).

**Figure 11:**
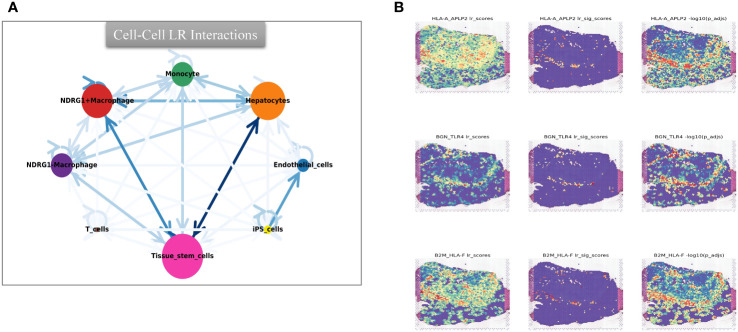
Intercellular cmmunication and ligand-rceptor analysis. **(A)** Intercellular communication at the ST level. **(B)** Ligand-receptor analysis at the ST level, including HLA-A_APLP2, BGN_TLR4, and B2M_HLA-F.

Furthermore, through scoring the co-expression of ligand-receptor pairs, we discovered a close association between NDRG1+Macrophages, liver-type cells, and tissue stem cells. Notably, three immunologically relevant ligand-receptor pairs, including major histocompatibility complex, class I, A-amyloid beta precursor-like protein 2 (HLA-A_APLP2), biglycan-toll-like receptor 4 (BGN_TLR4), and β2 microglobulin-human leukocyte antigen-F (B2M_HLA-F), were significantly co-expressed in the tumor centers and at the junction of tumors and normal tissues (**Figure 11B**). This finding highlights the existence of cellular communication between different cell types at the spatial transcriptional level. Specifically, tissue stem cells (defined as tumor cells through tissue sections) exhibited close communication with NDRG1+Macrophages and hepatocytes (**Figure 11A**).

### 2.10 Prognostic gene expression

**Figure 12:**
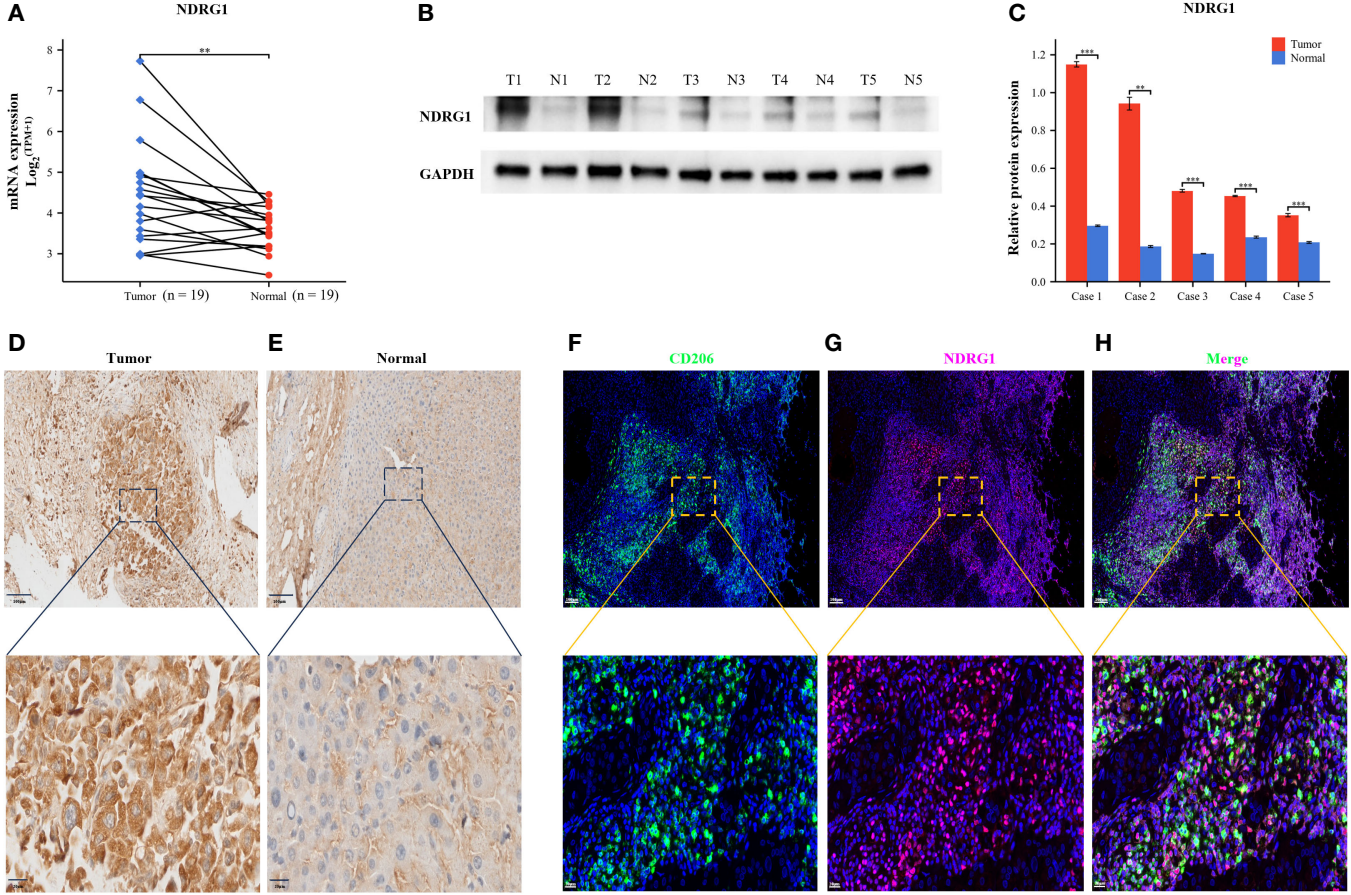
Validation of NDRG1 Expression in Hepatocellular Carcinoma. **(A)** NDRG1 mRNA expression profile in Patients with Hepatocellular Carcinoma. **(B)** NDRG1 protein expression profile in Patients with Hepatocellular Carcinoma. **(C)** Bar chart of relative expression levels of NDRG1 protein. **(D, E)** Immunohistochemical staining analysis of NDRG1 in tumor and normal liver tissues. **(F–H)** Immunofluorescence confocal microscopy analysis of CD206 and NDRG1 expression in HCC tissue.

To validate the robustness of our prognostic model, we performed an in-depth investigation into the potential relevance of NDRG1 in HCC. We meticulously examined the expression levels of this prognostic gene in human tissue samples. Through qRT-PCR analysis, we observed significantly elevated NDRG1 expression in tumor tissues (**Figure 12A**). Furthermore, to corroborate these findings at the protein level, we conducted Western blotting and IHC analyses, both of which confirmed the heightened protein expression of NDRG1 in tumor tissues (**Figures 12B–E**). These compelling results unequivocally demonstrate the upregulation of NDRG1 in HCC tissues, further emphasizing its potential significance in the context of HCC prognosis.

### 2.11 Co-expression of NDRG1, macrophages, and tumor cells

To elucidate the role of NDRG1 in the immune microenvironment, we collected specimens from patients with HCC. Multicolor immunofluorescence results demonstrated a significant elevation and co-localization of CD206 and NDRG1 expression in hepatocellular carcinoma tissues compared to paracancerous tissues (**Figures 12F–H**). These findings suggest that the high expression of NDRG1 in tumor tissues may induce the concentration of M2-type macrophages towards the tumor center, thereby facilitating immune escape and ultimately resulting in a poorer prognosis for patients with HCC.

## 3 Discussion

Recent developments in immunotherapy, molecularly targeted agents, and neoadjuvant chemotherapy have resulted in improved treatments for HCC. However, the prognosis for the long-term survival of patients continues to be poor. There is an urgent need for more sensitive and reliable prognostic indicators to monitor the progression of HCC and assess patient survival.

Disulfidptosis is a new method of controlling tumor cell development (8). To investigate disulfidptosis in HCC, we carried out a comprehensive analysis of ST, sc-RNA seq, and bulk RNA sequencing. First, we obtained 15 DRGs from Liu’s study and performed unsupervised consensus clustering, PCA, and UMAP. We then divided patients with liver cancer into two clusters and performed a survival analysis to find cluster 2, which had a poorer prognosis. Next, we used four algorithms to score disulfidptosis in patients with liver cancer and WGCNA to calculate the score for the blue modules most strongly related to disulfidptosis.

We then subjected the genes within the modules to multivariate Cox regression, LASSO regression, and univariate Cox regression analyses. Through these analyses, we identified six genes that are closely associated with liver cancer prognosis and constructed a prognostic model. Based on the median score, we categorized patients into low- and high-risk groups. The performance of the model was assessed using survival analysis, receiver operating characteristic analysis, and decision curve analysis, demonstrating its robustness and accuracy. In the study by Li (13), a prognostic model was constructed using different disulfidptosis modes to analyze differentially expressed genes. The AUC values under the ROC curve of the model were only 0.689 and 0.659 for 3-year and 5-year predictions, respectively. Additionally, in Yang’s study, although they utilized the WGCNA algorithm to construct a prognostic model, it only focused on identifying modules most correlated with clinical features and did not thoroughly analyze the expression patterns of disulfidptosis in liver cancer patients and the AUC values of the model’s 3-year and 5-year ROC curves are 0.739 and 0.685, respectively, which are also not as good as our model’s (14). In our study, for the first time, we calculated the disulfidptosis scores of liver cancer patients using four different algorithms from the ssGSEA package. We then identified the module with the highest correlation to the average disulfidptosis score using WGCNA (**Figure 5**). The prognostic model constructed based on this module exhibited better clinical predictive ability, with AUC values of 0.739, 0.757, and 0.712 for 1-year, 3-year, and 5-year predictions, respectively. Furthermore, we conducted an immune infiltration analysis and observed that the high-risk population exhibited upregulation in various cell types, including CLP, epithelial cells, iDC, osteoblasts, type 1 T helper (Th1) cells, and the type 2 T helper (Th2) cell pathway. Conversely, CD4 memory T cells, Th1 cells, Th2 cells, Macrophages_M1 cells, and neutrophils were more likely to infiltrate the high-risk groups. Notably, CD4 T cells have the ability to produce significant amounts of IL-22 cytokines (15), which have been implicated in driving HCC progression by promoting tumor cell proliferation (16). Additionally, cancer cells can influence memory CD4 T cells to express and release IL-1 in an IL-22-dependent manner, thereby facilitating tumor growth (17). Regulatory T cells (Tregs), a subset of immunosuppressive T cells, are commonly enriched in various cancer types and contribute to immune evasion by tumors. In the context of human breast cancer (BC), Tregs predominantly originate from naive CD4 T cells. The presence of Tregs is closely associated with an abundance of naive CD4 T cells, which serves as a prognostic indicator for poor outcomes in BC patients (18).

The lack of effector memory T cells (CD8 Tem) and central memory T cells (CD4 Tcm) explains immune incompetence and exhaustion in high-risk patients (19). Interestingly, high-risk patients are infiltrated with a higher number of M1, Th1, and Th2 macrophages. M1 macrophages secrete multiple inflammatory factors to sustain a prolonged inflammatory environment and recruit and initiate T cells early in tumor progression (20). We noted that high-risk patients were more prone to immune escape and immune rejection by TIDE analysis. We derived some potential immunotherapy targets by analyzing the relationship between the immune checkpoints and risk score.

Research has revealed that hepatocytes and macrophages frequently signal MIF and SPP1 to associate with other cell types. SPP1 is a sialic acid-rich chemokine-like glycoprotein that is overexpressed in a variety of cancers, including pancreatic cancer (21). Studies have demonstrated that an interaction between CD44 and SPP1 induces cell signaling and modulates tumor cell activation, motility, and adhesion, resulting in cancer progression and metastasis (22). MIF acts as a critical player in cell proliferation, tumorigenesis, and metastasis. MIF can activate the PI3K and MAPK pathways and modulate apoptosis, differentiation, proliferation, cell survival, and cancer progression (23). Midkine (MK), a cancer mediator that is highly expressed in a wide range of human malignancies, modulates cell growth, survival, migration, metastasis, and angiogenesis (24).

We performed scRNA-seq and spatial transcriptional analysis and found that the disulfidptosis score was higher in tumor cells and endothelial cells. NDRG1 was barely expressed in the normal tissues and highly expressed in the macrophages of metastatic lymph nodes, portal vein tumor thrombus (PVTT), and primary tumors. NDRG1 may promote tumor progression by affecting macrophage differentiation, as observed by pseudotime analysis, and is mostly involved in immune and oncogenic pathways. We validated this via ST.

NDRG1, commonly referred to as a metastasis suppressor protein, is expressed across various tumor types. This intracellular protein is composed of 394 amino acids, weighs 43 kD, and exhibits multiple isoforms (25). NDRG1 is actively involved in various cellular processes, such as DNA repair, immunity, and stress response. Additionally, NDRG1 plays pleiotropic roles depending on the type of cancer (26). In recent years, cancer immunotherapy has made significant progress, providing new opportunities for the treatment of liver cancer. However, the immune tolerance characteristics of the liver and the immunosuppressive tumor microenvironment (TME) in HCC have collectively hindered the development of effective anti-tumor immune responses against HCC. The presence of an immunosuppressive TME in liver cancer may be attributed to the accumulation of cells with negative regulatory immune activity, such as M2-polarized tumor-associated macrophages (27). Research has shown that tumor-associated macrophages (TAMs) in the tumor microenvironment are primarily composed of M2-type macrophages, which promote the expression of IL-1α, IL-1β, VEGF-A, and VEGF-C, thereby facilitating tumor growth and tumor angiogenesis/lymphangiogenesis (28). Additionally, several studies have confirmed that NDRG1 is highly expressed in macrophages within the tumor microenvironment (29). Observations have been made of a significant decrease in the serum levels of macrophage colony-stimulating factor (M-CSF) and macrophage-related cytokines in NDRG1 knockout mice (30). The deficiency of NDRG1 has been shown to attenuate the differentiation of macrophage lineage cells, leading to a suppression of bone remodeling and inflammatory angiogenesis (30). Mechanistically, NDRG1 interacts with the orphan nuclear receptor Nur77 and inhibits the transcriptional activity of NF-κB (nuclear factor Kappa B) (31). However, the loss of NDRG1 activates the NF-κB pathway, leading to the induction of epithelial-mesenchymal transition in prostate cancer (32). In their study, Chang et al. found that NF-κB activity is typically upregulated in classical M1 macrophages, while M2 macrophages are believed to have lower NF-κB activity and exhibit strong immunosuppressive effects (33). These findings indicate that NDRG1 may regulate macrophage polarization through the NF-κB pathway, ultimately leading to immune evasion. CD206, also known as an alternative activated macrophage marker, is a membrane glycoprotein expressed on the surface of macrophages, particularly M2 macrophages (34). Our multicolor immunofluorescence results have revealed a significant elevation and co-localization of CD206 and NDRG1 in hepatocellular carcinoma tissues. This further supports the close relationship between NDRG1 expression and macrophage differentiation. Tumor-associated macrophages (TAMs) account for more than 50% of all cells in tumors and play a crucial role as immune cells within the tumor microenvironment (35). Therapeutic strategies that incorporate or target TAMs have emerged as a promising and novel approach for anticancer therapy (36). On the other hand, one of the challenges in immunotherapy is the presence of immune suppressor cells in the tumor microenvironment, which can counteract the immune system’s attack on tumor cells. Research has found that tumor cells also induce immune tolerance by manipulating cells of the innate immune system, including polarizing macrophages into tumor-friendly M2 phenotypes and neutrophils into N2 phenotypes (37). Based on the findings from these studies, combined with our own research results, we speculate that NDRG1 may have a potential role in enhancing the efficacy of immunotherapy and reducing immunotherapy resistance. We aim to advance gene diagnosis and gene therapy into the early stages of cancer treatment, discovering more effective combination or sequential treatment strategies. Through future clinical research, we hope to gradually refine prognostic models to identify high-risk patients with poor prognosis in HCC. Targeted gene testing will be conducted on high-risk patients, and gene technology will be utilized for personalized treatment, either through specific knockout of NDRG1 or the design of NDRG1-targeted inhibitors. This approach may potentially reduce the M2/M1 ratio of tumor-associated macrophages in the tumor microenvironment, thereby preventing immune escape of cancer cells and improving the efficacy of immunotherapy and patient prognosis. However, further experimental validation is needed to confirm this hypothesis and extend it to other types of cancer.

In terms of limitations, our research lacks clinical data to evaluate the correlation between NDRG1 and immune therapy response as well as survival rates. Furthermore, the specific mechanisms underlying the interaction of NDRG1 with target genes and downstream signaling events require further investigation. These gaps will impede the translation of our findings into clinical applications, limiting the potential to provide valuable insights for personalized treatment and patient stratification.

In summary, the immediate research priorities following from these findings would involve further mechanistic studies, validation in preclinical models, identification of therapeutic targets, exploration of combination therapies, and clinical translation. These efforts could potentially lead to the development of novel therapeutic strategies for improving immune responses and treating HCC.

## 4 Conclusion

Our study provides the first comprehensive analysis of a disulfidptosis pattern in HCC in a large sample of the transcriptome, single-cell transcriptome, and spatial transcriptomics levels. We constructed a novel putative model that suggests high expression of the key factor NDRG1 may contribute to macrophage polarization, infiltration into the tumor center, and ultimately lead to a poor prognosis.

## 5 Materials and methods

### 5.1 Data acquisition and preprocessing

We obtained clinical information and bulk RNA sequencing data of HCC patients from various sources. The GEO14520, GSE144269, International Cancer Genome Consortium-Liver Cancer in Japan (ICGC-LIRI-JP), and TCGA Liver Hepatocellular Carcinoma (TCGA-LIHC) datasets were downloaded from the Gene Expression Omnibus database (https://www.ncbi.nlm.nih.gov/geo/), the ICGC data portal (https://dcc.icgc.org/), and TCGA data portal (https://www.cancer.gov/tcga/), respectively. For single-cell RNA sequencing (scRNA-seq) data of HCC, we downloaded the GSE149614 and GSE166635 datasets from the GEO database (https://www.ncbi.nlm.nih.gov/geo/). The GSE166635 dataset contains tumor scRNA-seq data from two HCC patients, while the GSE146914 dataset includes four relevant sites from 10 patients with different stages of PVTT, primary tumor, non-tumor liver, and metastatic lymph node. To acquire spatial transcriptome (ST) information for HCC tissue sections, we accessed the Single-Cell Colorectal Cancer Liver Metastases (CRLM) Atlas web portal (http://www.cancerdiversity.asia/scCRLM). The DRGs were obtained from Liu’s article (https://doi.org/10.1038/s41556-023-01091-2). After addressing batch effects, we integrated the GSE14520 and ICGC-LIRI-JP datasets, resulting in the formation of metadata. This metadata was used as the training set, while the TCGA-LIHC and GSE144269 datasets served as independent validation sets. All of the bulk transcriptome data were transformed logarithmically and transformed to transcripts per million (TPM) before analysis.

### 5.2 Expression analysis of disulfidptosis related genes in pan-cancer

We investigated the expression patterns of DRGs across a diverse range of tumor types using the Gene Set Cancer Analysis dataset (GSCALite) (http://bioinfo.life.hust.edu.cn/web/GSCALite/). Specifically, we examined the genomic locations of CNV mutations in DRGs on the 23 chromosome pairs, as well as CNV mutations and SNV in liver cancer. To visualize these findings, we employed R (Version 4.2.0) to generate graphical representations for 15 selected DRGs. Furthermore, we retrieved the protein-protein interaction network of the DRGs from the STRING database (https://string-db.org/cgi/input.pl) and visualized it using Cytoscape 3.9.

### 5.3 Methylation and drug sensitivity of DRGs

We conducted an analysis of DNA methylation levels in the pan-cancer using the GSCALite website, specifically focusing on the DNA methylation levels of DRGs. Additionally, we investigated the correlation between mRNA expression and DRG methylation levels across different tumor types. Furthermore, we performed a drug sensitivity analysis of DRGs using two databases: the Cancer Therapeutics Response Portal (CTRP) and the Genomics of Drug Sensitivity in Cancer (GDSC) databases.

### 5.4 Unsupervised consensus clustering for DRGs on patients with hepatocellular carcinomas

To explore the different disulfidptosis patterns of HCC, we applied the “ConsensusClusterPlus” R package to determine the subgroups of patients with HCC based on DRGs. We also verified the discriminatory degree of the categorization using the UMAP and PCA dimensionality reduction. Then, we subjected clusters to a survival analysis by applying the “survival” R package.

### 5.5 Disulfidptosis score and the weighted gene co-expression network analysis for the disulfidptosis-related module

In our study on HCC, we initially utilized four scoring methods from the “GSVA” R package to assess the disulfidptosis status. The average value of these scores was then used to represent the disulfidptosis characteristics of liver cancer patients (38, 39). Subsequently, we employed the WGCNA method to identify gene modules that were highly correlated, as well as the interconnections between these modules and their associations with disulfidptosis scores. This analysis aimed to identify potential therapeutic targets or candidate biomarkers. To construct the gene co-expression network, we utilized the “WGCNA” R package and selected modules that exhibited the strongest correlation with disulfidptosis in HCC (39). Prior to the analysis, we pre-processed the sample data and removed any outliers. We then constructed a correlation matrix using the “WGCNA” R package. By determining the optimal soft threshold, we transformed the correlation matrix into an adjacency matrix and subsequently built a topological overlap matrix (TOM). Through hierarchical clustering based on the TOM dissimilarity metric, genes with similar expression patterns were grouped into gene modules using average association. The module that exhibited the strongest correlation with disulfidptosis was selected as the critical module for further analysis.

### 5.6 Construction and verification of the DRG-based prognostic signature

We constructed and verified the prognostic characteristics of patients with HCC for key module genes from the WGCNA that are closely associated with disulfidptosis. First, we screened the prognosis-associated genes in the metadata training set by applying univariate Cox regression (“survival” R package). We then conducted a multivariate Cox regression and least absolute shrinkage and selection operator (LASSO) regressions (“glmnet” R package) to minimize the candidate genes and create a prognostic signature. We calculated the risk score by multiplying the regression coefficient (b) from the multivariate Cox regression by a linear combination of gene expression levels. The risk score calculation formula is:


$$Risk\;Score=\sum_{i=1}^{n}Coefficient\left(\beta_{i}\right)*x_{i}$$

Per the median risk score, we classified patients with HCC into low- and high-risk groups. We then drew time-dependent ROC curves (“pROC” R package) and Kaplan-Meyer survival curves (“survival” and “survminer”) to detect the clinical model’s prognostic value. We validated the new model’s robustness and assessed the prognostic value with TCGA-LIHC and GSE144269. Using the Human Protein Atlas database (HPA) (https://www.proteinatlas.org), we compared the protein expression patterns of the signature HCC genes to normal tissue. We built a predictive nomogram model (“survival” and “rms” R packages) incorporating tumor Tumor, Node, Metastasis (TNM) pathologic staging to predict the one-, three-, and five-year OS probability of patients with HCC based on the multivariate Cox regression analysis results. In addition, we conducted a DCA to determine the model’s net clinical benefits on OS at one, three, and five years for patients with HCC.

### 5.7 Tumor immune infiltration and GSVA analyses

To assess the tumor microenvironment across different risk groups, we employed the xCell algorithm (40). Furthermore, we obtained exclusion scores, dysfunction scores, and TIDE scores from the TIDE website (http://tide.dfci.harvard.edu/) (41). In order to evaluate the response to immunotherapy in the high- and low-risk groups, we compiled a list of 40 ICGs based on the literature (42). We then conducted a correlation analysis between the risk scores and the ICGs using the “corrplot” R package. Additionally, we utilized the “GSVA” R package to examine the expression patterns of different risk profiles in the Kyoto Encyclopedia of Genes and Genomes (KEGG) signaling pathway. For this analysis, we retrieved the C2 (C2.cp.Kegg.v7.4.symbols.gmt) gene set from the Molecular Signatures Database and generated a heatmap to visualize the results.

### 5.8 Analysis of scRNA-seq data

First, we utilized the “Seurat” package to generate objects and performed quality control measures to filter out lower-quality cells. Specifically, we applied the following criteria: cells with fewer than 200 or more than 4000 expressed genes were excluded, and cells with more than 10% of unique molecular identifiers (UMIs) mapped to mitochondrial genes were also excluded. We retained only genes that were expressed in at least three cells. Next, we normalized the data and identified the top 3000 highly variable genes using the “FindVariableFeatures” function. Principal component analysis (PCA) was then performed on the scRNA-seq data using these 3000 genes. For visualization and clustering purposes, we retained the first 16 and 22 principal components for GSE149614 and GSE166635, respectively, and applied the UMAP algorithm. To address batch effects between samples, we employed the harmony method (v0.1.0) to remove these effects and integrate the Seurat objects into a single dataset. Subsequently, we performed cell clustering using the “FindClusters” function in the “Seurat” package, with a resolution parameter set to 0.7. To annotate the cells, we utilized the “singleR” package and CellMarker 2.0 (http://bio-bigdata.hrbmu.edu.cn/CellMarker).

### 5.9 Cell-cell interaction and pseudotime analyses at single-cell level

To investigate cell-cell interactions, we employed the “CellChat” R package (43) and utilized its “cellchat” function. Our analysis involved utilizing ScRNA-seq count files and cell type-specific markers as input data. Using this approach, we examined the expression of receptors in one cell type and ligands in another. By assessing the presence of ligand-receptor interactions, we quantified the enrichment of such interactions between pairs of cell types. This analysis provided insights into the extent of communication and signaling between different cell types. To evaluate the cell-type specificity of a particular ligand-receptor complex, we identified P-values based on the proportion of mean values greater than or equal to the actual mean. We utilized a P-value threshold of< 0.05 to select important cell-cell interactions.

In parallel, we employed a pseudotime analysis of the scRNA-seq data to measure the evolutionary trajectory of macrophages in GSE166635. This analysis entailed mapping the high-dimensional gene expression data onto a one-dimensional quantity called pseudotime. We inferred cell fates and revealed the cellular trajectories. We utilized the “Monocle” R packages (Version 2.26.0) (44, 45), which can provide insight into the cellular developmental trajectory but cannot accurately determine the origin and direction of this developmental process.

### 5.10 Spatial transcriptomics data analysis

We processed and visualized the ST data using the “Seurat” R package. To ensure data comparability, we integrated the ST data using the SCT approach and subsequently performed clustering of similar ST sites using PCA. Specifically, we established filtering criteria, including a gene count between 300 and 6000, a mitochondrial ratio below 15%, and the exclusion of genes expressed in fewer than 10 spots. Then, we used the sctransform normalization method and PCA dimensionality reduction is performed first, and then the top 20 dimensions are selected for clustering and umap dimensionality reduction. Cell clusters were annotated based on hematoxylin and eosin (HE) staining sections and genes exhibiting high variability within each cluster. The spatial expression of DRGs was visualized using the SpatialDimPlot function. Furthermore, we conducted deconvolution using the “spacexr” package and utilized the “spotlight” R packages to identify cell types at specific spatial spots. Subsequently, we employed the “scMetabolism” R package to evaluate the metabolic activity of the spatial transcriptional data on slices (46) Additionally, we utilized the Python “stlearn” package to visualize and analyze cell-cell interactions, as well as score the co-expression of ligand-receptor pairs in the tissue slice.

### 5.11 Human specimens

We obtained 19 HCC pairs and adjacent non-cancerous specimens from the Department of Hepatobiliary-pancreatic & Hernia Surgery at Guangdong Second Provincial General Hospital. The study was authorized by the Medical Research Ethics Committee of Guangdong Second Provincial General Hospital, and all of the participants provided written informed consent. Following specimen isolation, we rapidly froze the liver tissue in liquid nitrogen and stored it at a temperature of -80°C to ensure preservation and prevent degradation.

### 5.12 Quantitative reverse transcription polymerase chain reaction

We extracted the total RNA with Trizol reagent (Invitrogen, Carlsbad, CA, USA), and synthesized the cDNA through the ABI 7500 Fast System (Applied Biosystems, Rockville, MD, USA). We used α-Tubulin as the reference gene. The relative expression level of the relevant gene was 2- [(Ct of gene) – (Ct of α-tubulin)], in which Ct stands for the threshold cycle. Primer sequences for amplification were as below: NDRG1 (47), forward primer, 5’-CTGCACCTGTTCATCAATGC-3’ and reverse primer, 5’-AGAGAAGTGACGCTGGAACC-3’.

### 5.13 Western blotting

The HCC tissue samples were lysed using a radioimmunoprecipitation assay buffer containing 1% phenylmethylsulfonyl fluoride (PMSF, Beyotime, Shanghai, China). Western blotting was performed following a previously described protocol. Primary antibodies specific to NDRG1 (1:5000, T57079S, Abmart) and an anti-glyceraldehyde-3-phosphate dehydrogenase (GAPDH) antibody (1:5000, Proteintech) were used, with GAPDH serving as a control. The obtained results were subjected to semi-quantitative analysis using ImageJ software.

### 5.14 Immunocytochemistry

To evaluate the expression of NDRG1 protein, we performed immunohistochemistry (IHC) experiments. Fresh human tissues were fixed overnight in 10% formalin, followed by dehydration, embedding in paraffin, and sectioning. The sections were then dewaxed and hydrated accordingly. Antigen retrieval was performed using citrate, and peroxidase activity in liver samples was blocked with 3% H_2_O_2_. The primary antibodies against NDRG1 (1:500, T57079S, Abmart) were incubated overnight at 4°C. Subsequently, the slides were incubated with a secondary antibody at 37°C for one hour. A 3,3’-diaminobenzidine (DAB) color development kit was employed, followed by hematoxylin restaining. Finally, the slides were dehydrated, rendered transparent, and sealed with neutral treacle. We then viewed the slides under a microscope, and two experienced pathologists conducted double-blind readings to identify the staining intensity and the percentage of positive cells, which was scored as follows:< 5% was scored as 0, 5%-25% was scored as 1, 26%-50% was scored as 2, 51%-75% was scored as 3, and 76%-100% was scored as 4. Moreover, we assessed staining intensity as follows: 0, 1, 2, and 3 for colorless, light yellow, tan, and brown, respectively. Lastly, We acquired the final score by multiplying the staining intensity score by the percentage of positive cells. Scores of 0, 1-4, 5-8, and 9-12 were negative (-), weakly positive (+), positive (++), and strongly positive (++++), respectively.

### 5.15 Immunofluorescence

For immunofluorescence staining, we utilized a multiplex immunofluorescence staining kit (abs50012, absin, Shanghai, China) and followed the instructions provided by the manufacturer. Antibodies against NDRG1 (1:500, T57079S, Abmart) and CD206 (1:500, TD4149S, Abmart) were incubated at room temperature for one hour. Subsequently, the slides were incubated with anti-rabbit/mouse IgG conjugated with HRP for 15 minutes at room temperature, followed by incubation with fluorophore-conjugated tyramine molecules (PPD 650, PPD 570, or PPD 520) for 15 minutes. Finally, the nuclei were stained using DAPI.

### 5.16 Statistical analysis

We conducted data analysis and visualization using the R software (Version 4.2.0, https://www.r-project.org/) and Python software (Version 3.9.0, https://www.python.org/). To compare two groups and two or more groups, we employed the Wilcoxon rank-sum test and the Kruskal-Wallis test, respectively. Categorical variables were compared using Fisher’s exact test or the chi-square test. Differences in survival curves were assessed using the log-rank test. We performed a Spearman’s correlation test to determine the correlations between the two variables. Statistical significance was determined at a significance level of P< 0.05.

## Data availability statement

The datasets presented in this study can be found in online repositories. The names of the repository/repositories and accession number(s) can be found in the article/**Supplementary Material**.

## Ethics statement

The studies involving human participants were reviewed and approved by The Medical Research Ethics Committee of Guangdong Second People's Hospital. The patients/participants provided their written informed consent to participate in this study. Written informed consent was obtained from the individual(s) for the publication of any potentially identifiable images or data included in this article.

## Author contributions

TY: Data curation, Investigation, Methodology, Software, Writing – original draft. JLi: Formal Analysis, Writing – original draft. FL: Investigation, Software, Writing – original draft. JLe: Software, Supervision, Writing – original draft. SC: Resources, Visualization, Writing – original draft. ZM: Project administration, Visualization, Writing – original draft. PK: Investigation, Methodology, Software, Writing – original draft. QY: Funding acquisition, Investigation, Writing – original draft. JW: Conceptualization, Investigation, Writing – review & editing. YH: Conceptualization, Data curation, Writing – review & editing. JD: Conceptualization, Data curation, Formal analysis, Funding acquisition, Writing – review & editing. XZ: Conceptualization, Funding acquisition, Investigation, Methodology, Writing – original draft, Writing – review & editing.

## Supplementary material

Supplementary Figure 1 Validation of the putative model. **(A–D)** Unsupervised consensus clustering. TCGA-LIHC **(E)** We plotted the OS curves between the high-risk and low-risk groups based on the prognostic signature. **(F)** The time-dependent ROC curves. **(G)** DCA for the validation data. GSE144269 **(H)** We plotted the OS curves between the high-risk and low-risk groups based on the prognostic signature. **(I)** The time-dependent ROC curves. **(J)** DCA for the validation data.

Supplementary Figure 2 The forest plot shows the hazard ratios and 95% confidence intervals of the signature genes from the univariate Cox regression analysis.
