# Supplementary figures and images for: Integrative analysis of disulfidptosis and immune microenvironment in hepatocellular carcinoma: a putative model and immunotherapeutic strategies
Source: Front Immunol. 2024 Jan 3;14:1294677. doi: 10.3389/fimmu.2023.1294677 (PMC10791859; doi:10.3389/fimmu.2023.1294677)

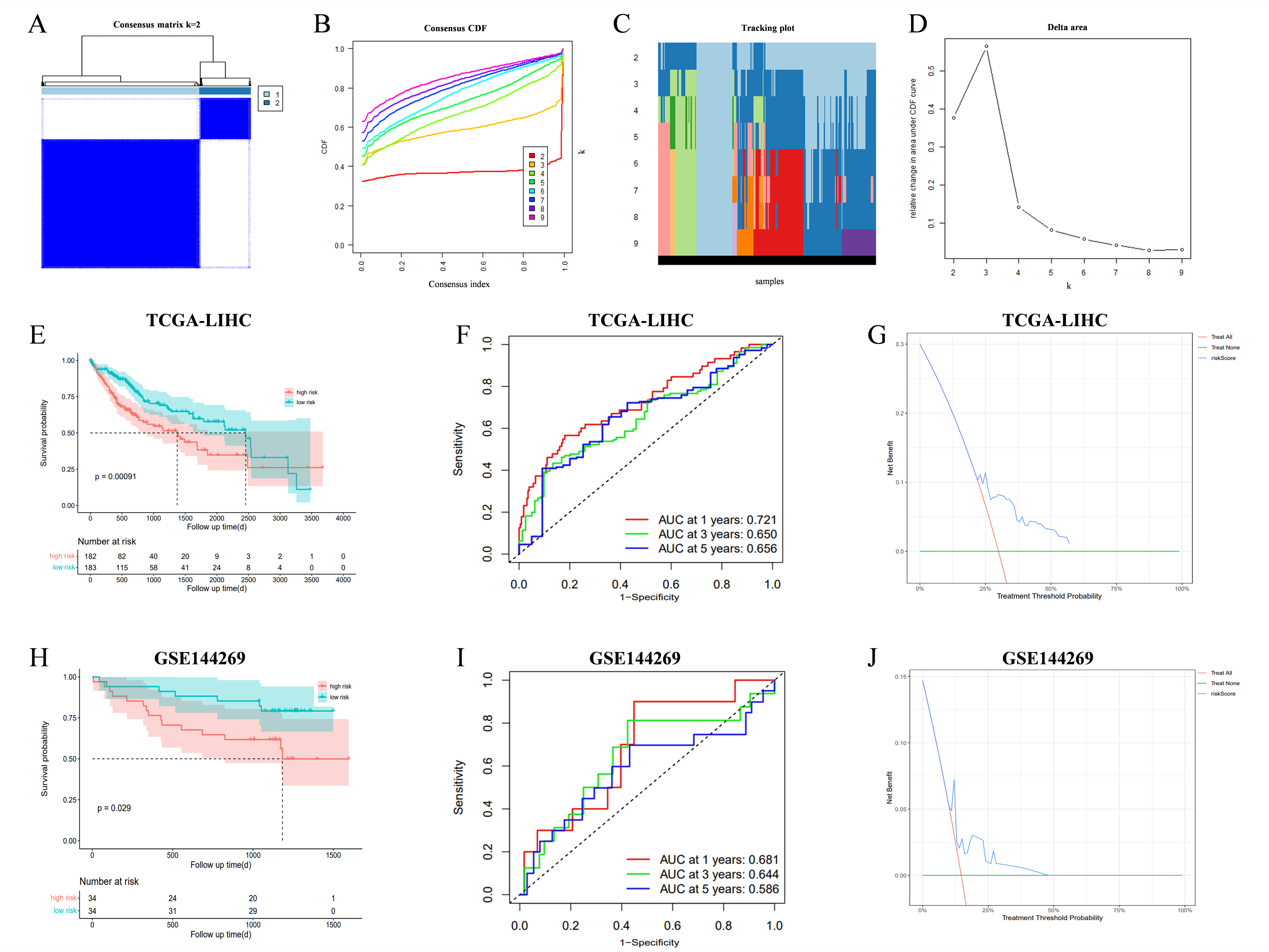

Supplement: Supplementary Figure 1 — Validation of the putative model. (A–D) Unsupervised consensus clustering. TCGA-LIHC (E) We plotted the OS curves between the high-risk and low-risk groups based on the prognostic signature. (F) The time-dependent ROC curves. (G) DCA for the validation data. GSE144269 (H) We plotted the OS curves between the high-risk and low-risk groups based on the prognostic signature. (I) The time-dependent ROC curves. (J) DCA for the validation data. [file Image_1.tif]

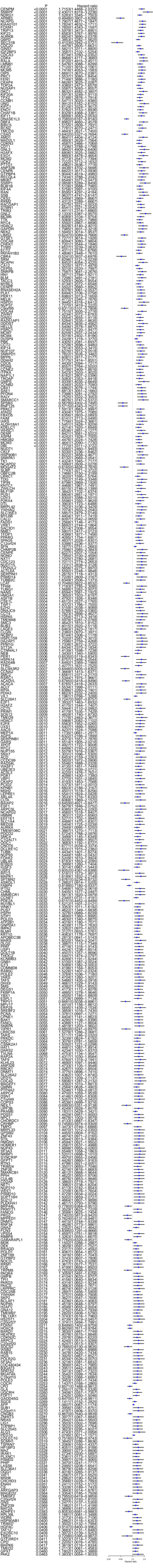

Supplement: Supplementary Figure 2 — The forest plot shows the hazard ratios and 95% confidence intervals of the signature genes from the univariate Cox regression analysis. [file DataSheet_1.pdf]
